# Human Aqueous Humor Levels of TGF-**β**2: Relationship with Axial Length

**DOI:** 10.1155/2014/258591

**Published:** 2014-05-20

**Authors:** Yan Jia, Dan-Ning Hu, Jibo Zhou

**Affiliations:** ^1^Department of Ophthalmology, Shanghai Ninth People's Hospital, Shanghai Jiaotong University, School of Medicine, 639 Zhizaoju Road, Shanghai 200011, China; ^2^Departments of Ophthalmology and Pathology, New York Eye and Ear Infirmary, New York, NY 10003, USA

## Abstract

*Purpose*. To analyze the relationship between transforming growth factor-beta 2 (TGF-**β**2) levels in the anterior chamber aqueous humor and axial length of patients with myopia. *Methods*. TGF-**β**2 was measured with the Luminex xMAP Technology by using commercially available Milliplex xMAP Kits. Sixty-five aqueous humor samples were collected during cataract or clear lens extraction surgery and TGF-**β**2 levels in these specimens were analyzed. According to the axial length, the samples were divided into three groups: A (AL ≤24 mm), B (24~29 mm), and C (AL ≥ 29 mm). *Results*. Aqueous humor samples were analyzed from subjects with an average age of 67.0 ± 11.7 years. Mean TGF-**β**2 concentration of all aqueous samples was 422.2 ± 258.8 pg/mL. TGF-**β**2 concentration in group C (543 ± 317 pg/mL) was significantly greater than that in group A (390 ± 212 pg/mL) and group B (337 ± 217 pg/mL). The concentration of TGF-**β**2 was positively correlated with axial length (*r* = 0.308, *P* = 0.013). *Conclusions*. TGF-**β**2 is likely to be acting as a critical factor in axial elongation and development of myopia.

## 1. Introduction

Myopia is a highly prevalent visual disorder, which may cause visual impairment and blindness. Excessive enlargement of the eye, predominantly in the axial dimension, results in the development of myopia in both humans and animal models. Myopia may result in a significantly increased risk of developing irreversible, sight-threatening pathology of the retina and choroid, like glaucoma [1], posterior scleral staphyloma [2], retinal detachment [3], and macular hole [4].

The development and progress of myopia are associated with marked thinning of the sclera at the posterior pole, which results in the elongation of axial length and the occurrence of myopia. The major metabolic changes in this process include reduced collagen synthesis, increased collagen degradation [5], reduced glycosaminoglycan synthesis [6, 7], altered integrin expression [8, 9], and influenced fibroblast to myofibroblast differentiation [10]. Experimental animal study and in vitro study indicated that these changes are critically mediated by changes in the levels of transforming growth factor-beta (TGF-*β*). Jobling's study first demonstrated the expression of all TGF-*β* isoforms in the mammalian sclera [11]. During myopia development in lens-induced guinea pigs, the activity of TGF-*β*2 of scleral desmocytes at the posterior pole was increased [12].

TGF-*β* is of primary importance in the regulation of extracellular matrix turnover [11]. Although five members of the TGF-*β* family have currently been identified, only TGF-*β*1, -*β*2, and -*β*3 have been detected in eyes [13, 14]. TGF-*β*2 is the main isoform of TGF-*β* in the eye and it is produced locally. TGF-*β*2 is a normal component of aqueous humor and the major isoform found in the sclera [15, 16]. Previous studies indicated that the change of expression of TGF-*β* is associated with the development of myopia [11, 12, 17, 18]. However, all of these results were obtained from in vitro study and experimental animal studies. To our best knowledge, very little was known on the changes of TGF-*β* in aqueous humor in myopia.

Aqueous humor is an important specimen that could be used to study the quantitative changes of growth factors in the eye. Therefore, we designed this experiment to study the aqueous levels of TGF-*β*2 in patients with myopia or cataract with different axial length and to investigate the relationship between TGF-*β*2 and axial elongation.

## 2. Materials and Methods

### 2.1. Patients and Samples

This study was an observational study on 65 patients with myopia or cataract. Exclusion criteria included prior intraocular surgery and patients with hypermature cataract. All patients were relatively healthy with no systemic diseases, such as kidney diseases, hematologic diseases, immune diseases, diabetes, or history of drug usage. Aqueous samples were collected from these patients during cataract or clear lens extraction surgery. This research was performed in accordance with the Helsinki Declaration. All patients signed an informed consent and the Institutional Review Board at the Shanghai Ninth People's Hospital Affiliated Shanghai Jiaotong University School of Medicine approved the study.

Axial length (AL) was measured with a Zeiss IOL-Master laser interferometer (Optical Biometry, IOL Master; Carl Zeiss Meditec AG, Jena, Germany). According to the AL, the 65 eyes were divided into three groups: group A (AL ≤ 24 mm), group B (AL between 24 and 29 mm), and group C (AL ≥ 29 mm).

Aqueous humor (0.1~0.2 mL) was collected at the beginning of operation as previously reported [19, 20]. After a corneal paracentesis, a 30-gauge needle on a tuberculin microsyringe was used to aspirate aqueous humor from the central anterior chamber. The aqueous humor samples were immediately stored under −80°C until analysis. All samples were protected from light.

### 2.2. Measurement of Levels of TGF-*β*2

All samples were assayed for TGF-*β*2 with the Luminex system (Luminex xMap Technology from Bio-Rad) by using commercially available Milliplex xMAP Kits (Millipore Corporation, Billerica, MA, USA) [21, 22]. This technology uses multiplexed microsphere-based immunoassays that apply flow cytometric resolution to measure spectrally distinct microspheres coupled with capture molecules and reporter fluorochromes bound to detection antibodies.

The assays were performed by following the manufacture's guidelines and were measured on a Bio-Plex 200 system. Each sample was measured in duplicate. A standard curve was traced for each test substance with the standard provided kit, according to the manufacturer's instructions. The same aqueous humor sample was measured in each plate for an interassay control. The detection limits were 6 pg/mL for TGF-*β*2.

### 2.3. Statistics

The results were expressed as mean ± standard deviation (SD) and analyzed statistically using a one-way ANOVA to compare the levels of aqueous humor TGF-*β*2 concentrations in different groups. The Pearson correlation test was used to analyze the relationship between aqueous axial length and TGF-*β*2 concentration. Independent-samples* t*-test was used to evaluate the means between males and females. A computer program (SPSS 22.0 for Windows; SPSS, Chicago, IL, USA) was used to do these analyses. A two-tailed *P* < 0.05 was considered to be statistically significant for this analysis.

## 3. Results

Aqueous humor samples were collected from 65 subjects, including 27 eyes with AL ≤ 24 mm (group A), 18 eyes with AL at 24~29 mm (group B), and 20 eyes with AL ≥ 29 mm (group C) (Table 1). The average age of the patients was 67.0 ± 11.7 years (mean ± SD). No statistically significant difference was found in gender (*χ* ^2^ = 1.220, *P* = 0.543) distribution among the three groups.

### 3.1. Concentration of TGF-*β*2

**Figure 1:**
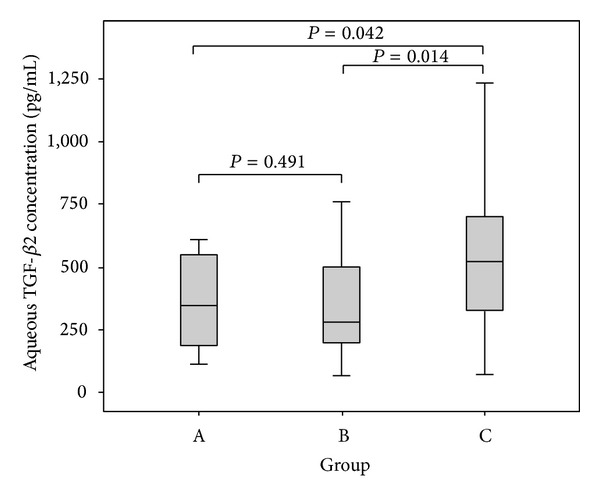
TGF-*β*2 concentration in three groups. TGF-*β*2 levels in the aqueous humor from group A (*n* = 27), group B (*n* = 18), and group C (*n* = 20) were measured with Luminex xMap Technology. One-way ANOVA with a post-hoc LSD test demonstrated that the differences of aqueous TGF-*β*2 concentration in three groups were statistically significant (*P*_AB_ = 0.491, *P*_AC⁡_ = 0.042, *P*_BC_ = 0.014, resp.).

Mean TGF-*β*2 concentration was 422.2 ± 258.8 pg/mL. TGF-*β*2 concentrations in groups A, B, and C were 390 ± 212 pg/mL, 337 ± 217 pg/mL, and 543 ± 317 pg/mL, respectively. The differences in the mean concentrations of TGF-*β*2 in three groups were statistically significant (*F* = 3.614, *P*_AB_ = 0.491, *P*_BC_ = 0.014, *P*_AC⁡_ = 0.042). TGF-*β*2 concentration in group C (with marked elongation of AL) was significantly greater than that in group A and group B, whereas no significant difference in TGF-*β*2 concentration could be found between group A and group B. (Table 1; Figure 1).

**Figure 2:**
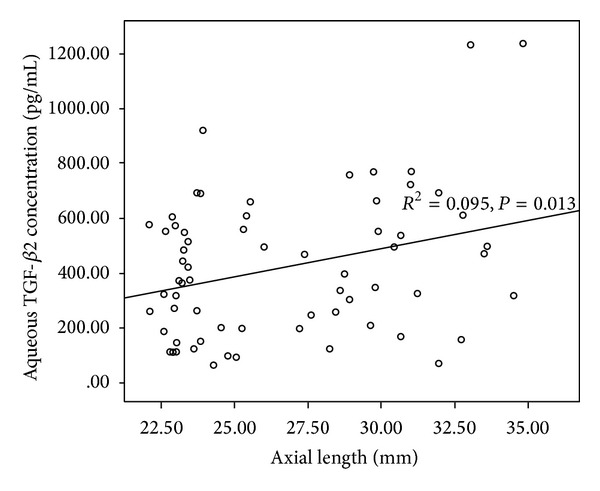
Correlation between axial length and aqueous TGF-*β*2.

The aqueous humor TGF-*β*2 concentration was significantly positively correlated with the AL (correlation coefficient *r* = 0.308, *P* = 0.013) in all subjects (Figure 2).

### 3.2. Relationship between TGF-*β*2 and Age

**Figure 3:**
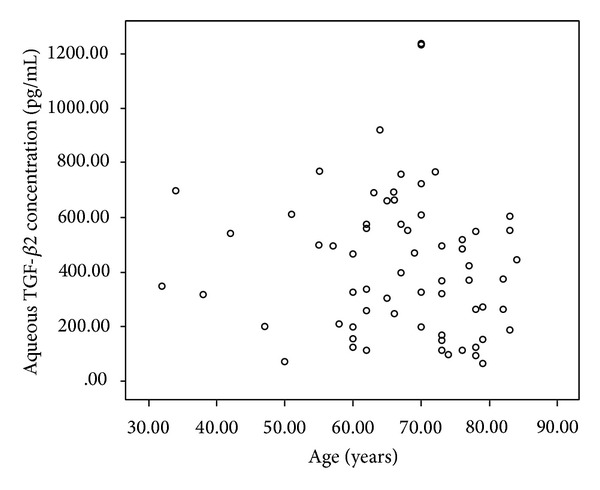
Correlation between age and aqueous TGF-*β*2.

No significant correlation was found between the age and TGF-*β*2 levels in the subjects of our study (*r* = −0.140, *P* = 0.290) (Figure 3).

### 3.3. Relationship between TGF-*β*2 and Gender

In our study, there was no significant difference in aqueous TGF-*β*2 concentration between males and females (*t* = 0.647, *P* = 0.520) (Table 1).

## 4. Discussion

In our study, TGF-*β*2 in aqueous humor could be detected and measured. TGF-*β*2 concentration in the aqueous humor was positively correlated with axial length significantly. In addition, the difference of the TGF-*β*2 level between group C (extremely high myopia with marked elongation of AL, more than 29 mm) and group A (AL ≤ 24 mm) was statistically significant. Similarly, TGF-*β*2 concentration in group C was significantly greater than that in group B.

Previously, researches have noted that TGF-*β* is an important factor in progression of growth and development of eyeball [23–25]. TGF-*β* has been shown to play a central role in organ development and homeostasis regulating cell proliferation, differentiation, and apoptosis [26]. Of the three isoforms of TGF-*β* (TGF-*β*1, TGF-*β*2, and TGF-*β*3) found in mammals, TGF-*β*2 is regarded as the major inform in the eye [11]. Elevated levels of TGF-*β*2 have been detected in the aqueous humor from glaucomatous eyes [14, 27], and reduced levels of active TGF-*β*2 have been detected in the aqueous humor of uveitic eyes [28].

Even though TGF-*β*2 is a key factor in the progression of myopia development and axial elongation, it has remained unclear what role would TGF-*β*2 play in the process. All three isoforms are capable of moderating collagen synthesis in scleral fibroblasts and TGF-*β*2 is the most potent one. In the current study, the concentration of TGF-*β*2 was positively correlated with axial length, indicating that changes of TGF-*β*2 levels are associated with the development of myopia. This is consistent with previous animal models reports that TGF-*β* was increased in myopic sclera tissue [12, 29–31] and also consistent with our previous study that documented that TGF-*β* could inhibit the growth of cultured human scleral fibroblasts [32].

Usually, one-millimeter extension of axial length represents diopter increased approximately −3.00 D [33], so the axial length of 29 mm means extreme high myopia with −15.00 D. A study on Han Chinese showed that TGF-*β*2 (rs7550232) polymorphisms were associated with myopia. The frequency of A allele and A/A of TGF-*β*2 (rs7550232) was higher in the control group than in the myopia group (*P* = 0.014), indicating that these genotypes of TGF-*β*2 (rs7550232) polymorphisms had a protective effect against the development of high myopia [34]. In our study, we demonstrated that the aqueous TGF-*β*2 levels were positively correlated with axial elongation of high myopia.

In the present study, age did not show any correlation with the aqueous humor concentration of TGF-*β*2. However, some studies on glaucoma patients reported that the TGF-*β*2 concentration in aqueous humor from anterior chamber decreases with age [35, 36]. The difference in results may be caused by different subjects and the sensitivity of detection methods.

In summary, different from previous studies, the object of our research is human aqueous humor rather than the sclera or other ocular tissues, and in this study we found that TGF-*β*2 was increased in the eyes with excessive elongation of axial length. Continued study of various growth factors and their functions, such as TGF-*β*2, may be helpful for the understanding of underlying pathophysiological changes in the development of myopia.

## Figures and Tables

**Table 1:** Main characteristics of 65 patients with cataract or myopia.

Variant	Total	Group A	Group B	Group C
Number	65	27	18	20
Age (years)	67.0 ± 11.7	74.3 ± 7.3	65.4 ± 5.8	58.3 ± 14.2
Gender (male/female)	29/36	11/16	10/8	8/12
Axial length (mm)	26.7 ± 3.8	23.1 ± 0.5	26.7 ± 1.7	31.6 ± 1.7
TGF-*β*2 concentration (pg/mL)	422.2 ± 258.8	389.8 ± 212.3	337.3 ± 211.6	542.5 ± 316.8

Sixty-five aqueous humor samples were collected from patients with myopia or cataract during cataract or clear lens extraction surgery. According to the AL, the samples were divided into three groups: group A: AL *≤  *24* *mm, group B: AL 24–29 mm, and group C: AL *≥  *29 mm.
